# Neural Network-Based System for Rapid Diagnostic Decision Support Using FLIM Data: Segmentation and Classification in Liver, Skin, and Pancreatic Tissues

**DOI:** 10.3390/ijms27188162

**Published:** 2026-09-14

**Authors:** Ilya Shchechkin, Svetlana Rodimova, Nikolai Bobrov, Vadim Elagin, Polina Ermakova, Artem Mozherov, Aleksandra Kashina, Vladislav Shcheslavskiy, Vladimir Zagainov, Elena Zagaynova, Daria Kuznetsova

**Affiliations:** 1 Institute of Experimental Oncology and Biomedical Technologies, Privolzhsky Research Medical University, Nizhny Novgorod 603950, Russia; nikolay_bobrov15@mail.ru (N.B.); elagin.vadim@gmail.com (V.E.); bardina-polina@mail.ru (P.E.); almele@yandex.ru (A.K.); shehes@yahoo.com (V.S.); ezagaynova@gmail.com (E.Z.); daria.s.kuznetsova@gmail.com (D.K.); 2 Institute of Information Technology, Mathematics and Mechanics, N.I. Lobachevsky Nizhny Novgorod National Research State University, Nizhny Novgorod 603950, Russia; 3 Volga District Medical Centre of Federal Medical and Biological Agency, Nizhny Novgorod 603000, Russia; 4 Institute for Regenerative Medicine, I.M. Sechenov First Moscow State Medical University, Moscow 119991, Russia; atemmozherov@gmail.com; 5 Nizhny Novgorod Regional Clinical Oncological Dispensary, Nizhny Novgorod 603126, Russia; zagainov@gmail.com; 6 Lopukhin Federal Research and Clinical Center of Physical-Chemical Medicine of Federal Medical Biological Agency, Moscow 119435, Russia

**Keywords:** FLIM, foundational models, neural network, liver, skin, pancreas

## Abstract

Fluorescence lifetime imaging microscopy (FLIM) is a powerful tool for analyzing metabolic changes by measuring the fluorescence lifetime of endogenous fluorophores such as the reduced form of nicotinamide adenine dinucleotide (phosphate) (NAD(P)H), enabling differentiation between normal and pathological states in diverse tissues. However, traditional manual FLIM data processing suffers from several drawbacks, including subjective selection of regions of interest, variability in analysis parameters, time-consuming procedures, and inherent human bias. To address these limitations, we developed an automated pipeline that leverages multiple pre-trained foundational models—SAM 2, CellposeSAM, LACSS, and CellSAM—for rapid, intensity-based segmentation and subsequent classification of the FLIM parameters in liver, pancreas, and skin tissues. Our comparative analysis identified the most suitable models for each tissue type and, crucially, enabled accurate discrimination between normal and pathological conditions. SAM 2 and LACSS demonstrated stable segmentation performance across both healthy and diseased tissues, though with distinct strengths: SAM 2 excelled at segmenting large structures (e.g., entire pancreatic islets of Langerhans, or very large cells), whereas LACSS was more effective for small structures. In contrast, CellposeSAM and CellSAM yielded less consistent segmentation overall but achieved notably high efficiency in specific cases. These findings establish a foundation for the rapid, automated discrimination of normal versus pathological tissues, offering a robust complement to existing clinical methods and to FLIM workflows for research.

## 1. Introduction

Fluorescence lifetime imaging microscopy (FLIM) is a powerful tool, with high spatiotemporal resolution, for analyzing the metabolic state of biological samples [1,2]. The fluorescence lifetime is a robust parameter in molecular imaging because it is sensitive to both fluorophore conformation and the local microenvironment [3,4,5,6]. This sensitivity indicates that FLIM provides a promising approach for rapidly distinguishing between normal and pathological states in tissues and organs.

Based on the intrinsic parameters of the fluorescence decay process and their derivatives, an effective classification of various tissue conditions has been demonstrated [7,8,9]. In this context, it is promising to develop an automated classification system for the FLIM data from each type of tissue to identify its pathological conditions, thereby providing a rapid decision support system.

FLIM has proven effective in identifying metabolic changes associated with various pathological states, including liver disease, diabetes, and cancer [7,10,11,12,13,14,15,16,17,18]. However, manual analysis of FLIM data introduces significant challenges that compromise accuracy and reproducibility:

1. Subjectivity in Thresholding: Manually defining intensity thresholds to separate signal from background is inconsistent, even for the same analyst over time.

2. Selection Bias: Manually drawing regions of interest (ROIs) can unconsciously favor areas that confirm a hypothesis, thereby undermining statistical objectivity.

3. Lack of Standardization: Without precise, documented protocols, reproducing and comparing results across laboratories is difficult.

4. Low Throughput: Processing large or complex datasets manually is time-consuming and labor-intensive.

These limitations underscore the urgent need for automated, standardized computational solutions. Advances in machine and deep learning now enable the consistent automation of segmentation, curve fitting, and quantitative analysis of FLIM data, promising to enhance the speed, simplicity, and reliability of this technique [19,20,21,22,23].

In this paper, we applied and compared four of the most commonly used foundational models for automatic semantic segmentation—SAM 2 (the segment anything model, a general-purpose foundation model) [24], CellposeSAM (a model that adapts the pre-trained transformer backbone of the SAM foundation model to the Cellpose [25] framework) [26], LACSS (the location assisted cell segmentation system, a model for point-supervised single-cell segmentation that uses a collaborative knowledge-sharing approach) [27] and CellSAM (uses a transformer-based object detector, CellFinder [28], to generate bounding box prompts for SAM to create instance segmentations) [29]. We also developed an algorithm for the automatic processing of complex FLIM datasets.

Comparative analysis of the different segmentation algorithms was carried out on fluorescence time-resolved images. Each algorithm possesses distinct advantages and disadvantages. We evaluated their performance on both normal tissues, which are straightforward to segment, and on pathological tissues, whose complex structures present significant segmentation challenges.

When developing the FLIM data analysis algorithm, we decided to use the results of FLIM data analysis based on commercial software, SPCImage (Becker & Hickl GmbH, Berlin, Germany), using neural networks and machine learning algorithms to automatically select objects of interest to us. Thus, the resulting algorithm combines both reliable SPCImage accuracy and independent tissue segmentation.

The developed classification algorithms can be translated into clinical practice to accelerate high-accuracy differentiation of normal and pathological conditions of tissues.

Our results offer the opportunity to determine further suitable algorithms and to develop our own additional solutions for processing FLIM images based on Python.

## 2. Results

To evaluate the metabolic state of the samples, we utilized FLIM. The resulting fluorescence decay curves were analyzed using bi- and tri-exponential fitting models. In this section, t1, t2 and t3 represent the fluorescence lifetime components for free, protein-bound and phosphorylated NAD(P)H, respectively. Their corresponding relative amplitudes (fractional contributions) are denoted by a1, a2 and a3. To provide a comprehensive metric for the overall decay kinetics, the amplitude-weighted mean fluorescence lifetime, tm, was calculated as a1/t1 + a2/t2 + a3/t3.

### 2.1. Characteristics of Segmentation Foundational Models

Based on the literature sources studied, we have compiled a comparative table that presents the characteristic features of the segmentation models used in the work (Table 1). Our subsequent emphasis was placed on assessing the relative quality, speed and comparability of segmentation provided by the selected models.

### 2.2. Segmentation Quality and Speed

#### 2.2.1. Quality

The analysis revealed notable variations in segmentation performance among the foundational models. Specifically, the SAM 2 and LACSS models produced comparable object counts for both healthy and pathological samples. In contrast, the CellSAM and cellposeSAM models exhibited a reduction in the number of segmented objects identified within pathological images. The LACSS model was characterized by a substantial degree of over-segmentation, frequently partitioning single objects into multiple discrete segments. Conversely, isolated instances of under-segmentation—where several objects were merged into a single segment—were observed for the SAM 2, CellSAM, and CellposeSAM models. The LACSS, CellposeSAM, and SAM 2 models detected the highest number of objects, whereas CellSAM detected the fewest.

When assessing the dependence of image segmentation quality on tissue pathology, we showed that, in the liver, pathology decreases the quality of segmentation. By contrast, pathology does not change the segmentation accuracy for images of skin and pancreatic (islets of Langerhans).

**Figure 1:**
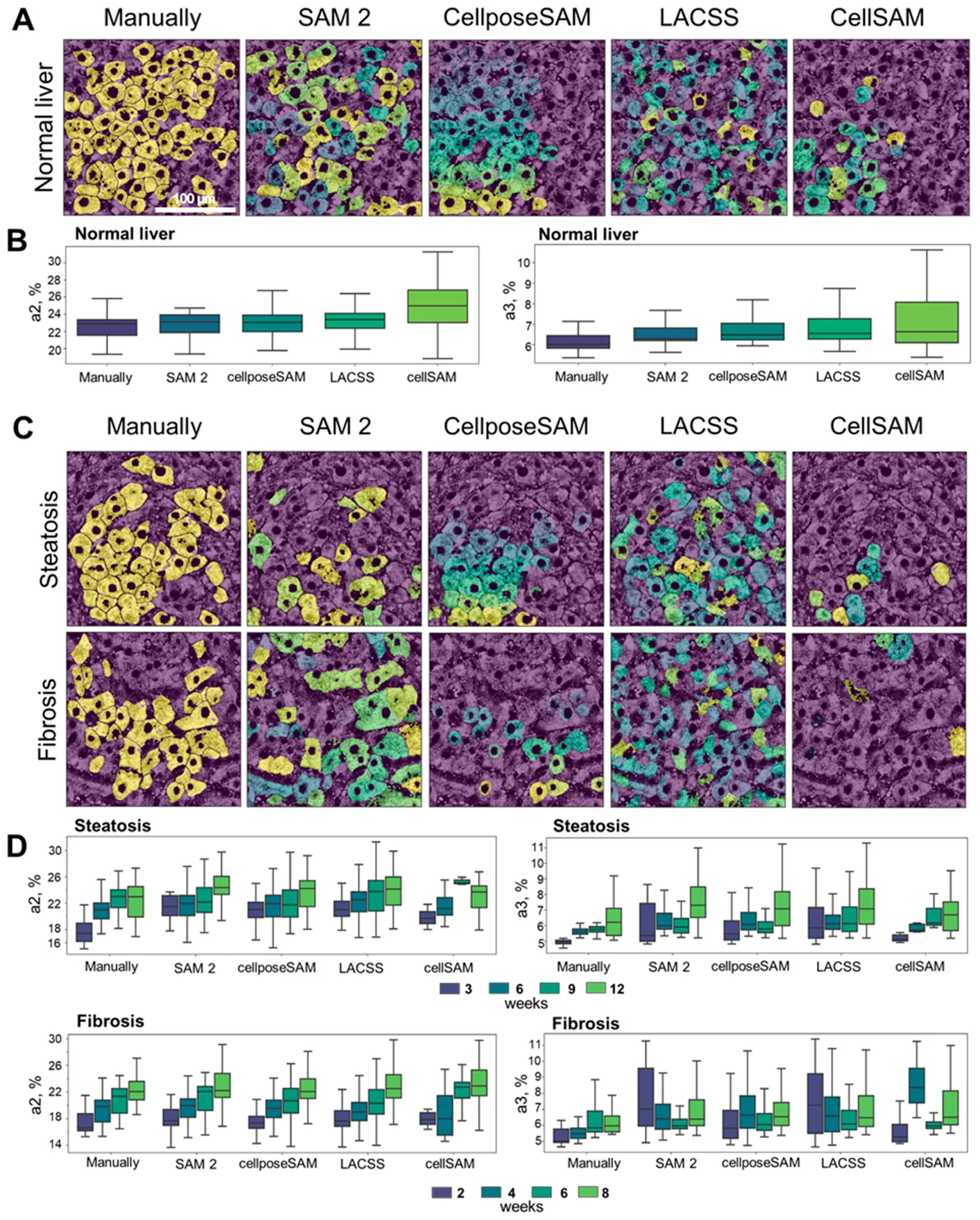
Comparative analysis of the accuracy and comparability of segmentation models for normal and pathological liver tissue. (**A**)—intensity images, with comparison of cell masks of normal liver tissue; the color of the cell in the image distinguishes individual cells; (**B**)—comparison of a2 and a3 values for various segmentation methods for normal liver tissue; (**C**)—intensity images, with comparison of cell masks of pathological liver tissue (steatosis and fibrosis); (**D**)—comparison of a2 and a3 values for the various segmentation models for pathological liver tissue (undergoing development of either steatosis or fibrosis).

Segmentation quality analysis for the liver showed that the CellposeSAM and LACSS models provided the most consistently high relative accuracy for normal tissues, with Recalls of 0.644 and 0.703 for CellposeSAM and LACSS, respectively, while offering moderate relative accuracy for pathological liver tissue, with respective Recalls of 0.463 and 0.679 for CellposeSAM and LACSS. The CellSAM model showed the lowest Recall values of 0.25 and 0.181 for normal and pathological liver tissue, respectively. The SAM 2 model had the least stable accuracy, with high standard deviation values of 0.306 and 0.255 for normal and pathological tissue, respectively, while the corresponding Recall values were 0.51 and 0.543. The results are shown in Figure 1A,C and Table 2 and Table S1.

**Figure 2:**
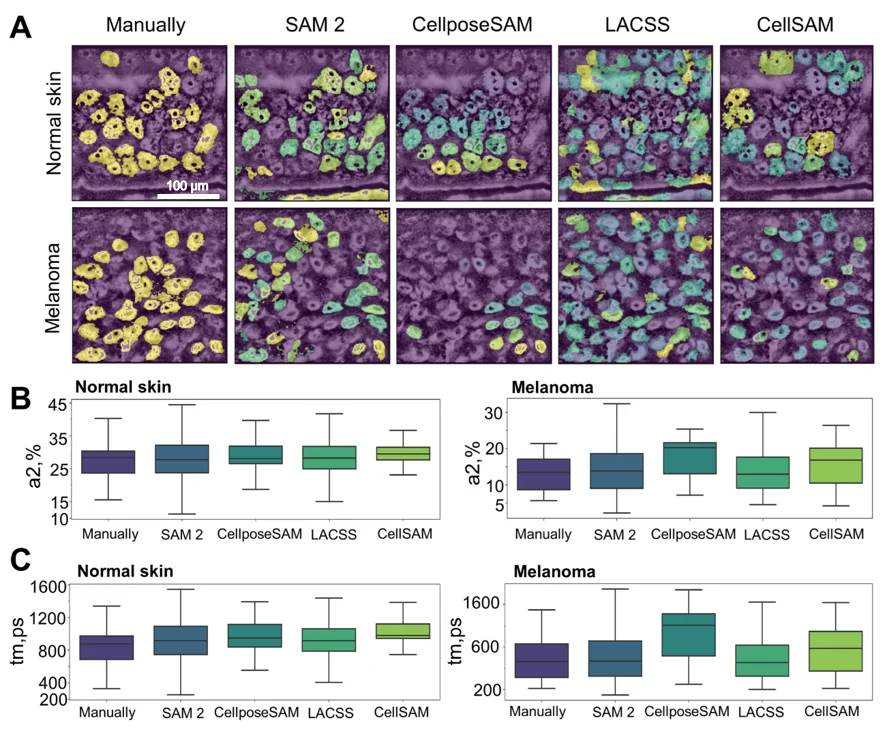
Comparative analysis of the accuracy and comparability of segmentation models for normal and melanoma skin tissue. (**A**)—intensity images, with comparison of cell masks of normal and pathological skin tissue; the color of the cell in the image distinguishes individual cells; (**B**)—comparison of a2 values for the various segmentation methods for normal and melanoma skin tissue; (**C**)—comparison of FLIM parameter tm values for the various segmentation methods for normal and melanoma skin tissue.

For skin tissue, the LACSS model showed the highest accuracy and stability (0.711 ± 0.068 for the normal state, 0.822 ± 0.075 for melanoma), while the SAM 2 model showed moderate accuracy (0.341 ± 0.146 for the normal state, 0.398 ± 0.087 for melanoma). The CellposeSAM (0.429 ± 0.222 for the normal state, 0.17 ± 0.126 for melanoma) and CellSAM (0.271 ± 0.352 for the normal state, 0.413 ± 0.198 for melanoma) models showed low accuracy or stability. The results are presented in Figure 2A and Table 2 and Table S2.

**Figure 3:**
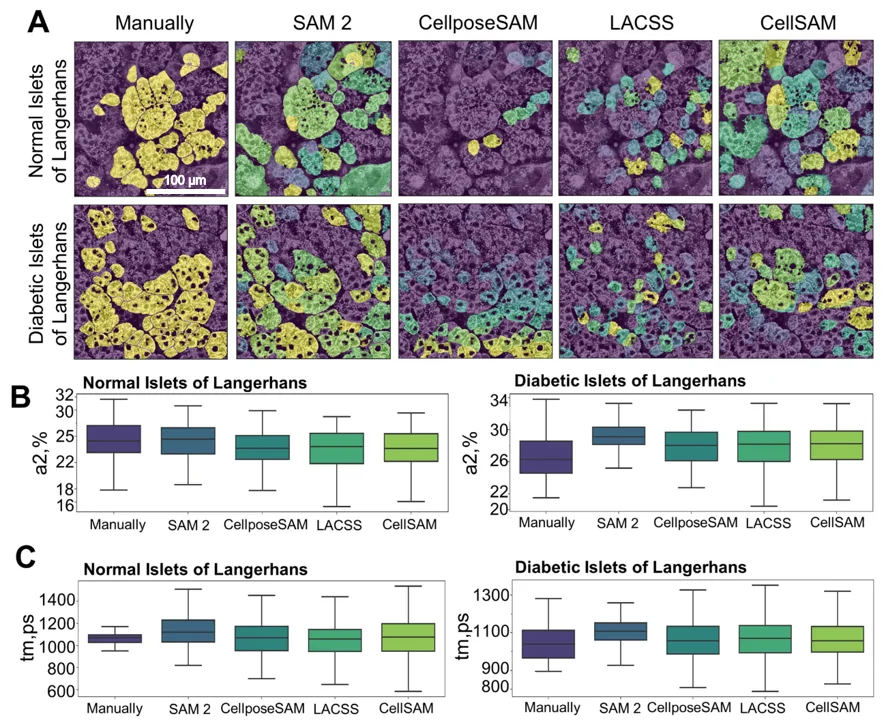
Comparative analysis of the accuracy and comparability of segmentation models for normal and diabetic pancreatic tissue. (**A**)—intensity images, with comparison of cell masks of normal and diabetic pancreatic tissue; the color of the cell in the image distinguishes individual cells; (**B**)—comparison of FLIM parameter a2 values for the various segmentation methods for normal and diabetic pancreatic tissue; (**C**)—comparison of FLIM parameter tm values for the various segmentation methods for normal and diabetic pancreatic tissue.

For pancreatic tissue (islets of Langerhans) analysis of the segmentation quality showed that the SAM 2 (0.856 ± 0.032 for normal tissue, 0.665 ± 0.163 for diabetes) and CellSAM (0.937 ± 0.015 for normal tissue, 0.743 ± 0.087 for diabetes) models had the highest accuracy, while the LACSS (0.609 ± 0.053 for normal tissue, 0.566 ± 0.059 for diabetes) model was slightly lower in accuracy. The CellposeSAM model had both the lowest accuracy and stability (0.446 ± 0.381 for normal tissue, 0.463 ± 0.105 for diabetes). The results are shown in Figure 3A and Table 2 and Table S3.

#### 2.2.2. Speed

The highest segmentation speed was shown for the CellposeSAM model, averaging 5.148 ± 0.243 s, contrasting with the lowest speed at 55.59 ± 2.027 s for the SAM 2 model, while for the LACSS and CellSAM models the segmentation process took 10.263 ± 5.074 and 6.63 ± 3.195 s, respectively.

### 2.3. Segmentation Comparability

Comparative analysis of the FLIM parameters for pathological liver tissue based on segmentation by the various foundational models shows that CellposeSAM had the highest accuracy and stability, with an average accuracy of 0.611 ± 0.1 and 0.74 ± 0.054 for steatosis and fibrosis, respectively. The SAM 2 model provided high comparative accuracy for fibrosis, with an average accuracy of 0.741 ± 0.069 and a moderate average accuracy of 0.579 ± 0.164 for steatosis. The LACSS model provides relatively moderate comparative accuracy for both steatosis (0.573 ± 0.111) and fibrosis (0.692 ± 0.061). The CellSAM model had the lowest comparative accuracy values at 0.44 ± 0.02 for steatosis and 0.438 ± 0.068 for fibrosis. The results are shown in Figure 1C,D and Table 2 and Tables S4 and S5.

For skin, it was shown that the LACSS model had the highest comparability (0.84 for normal skin and 0.89 for melanoma) with manually obtained results. CellSAM for normal skin (0.558) and CellposeSAM for melanoma (0.504) had the lowest levels of comparability with the results of manual analysis. For the pancreas (Islets of Langerhans), it was shown that CellposeSAM, LACSS and CellSAM provide results that are approximately equal and highly comparable with manual analysis (~0.75). While being the lowest, SAM 2 also showed quite a high degree of comparability with manual analysis (0.683 and 0.523 for the normal state and in diabetes, respectively). The results are shown in Figure 2B,C and Figure 3B,C and Table 2 and Table S6.

While we found a correlation between the values for both comparability and accuracy for liver and skin tissues, no such correlation was demonstrated for pancreatic tissue.

**Figure 4:**
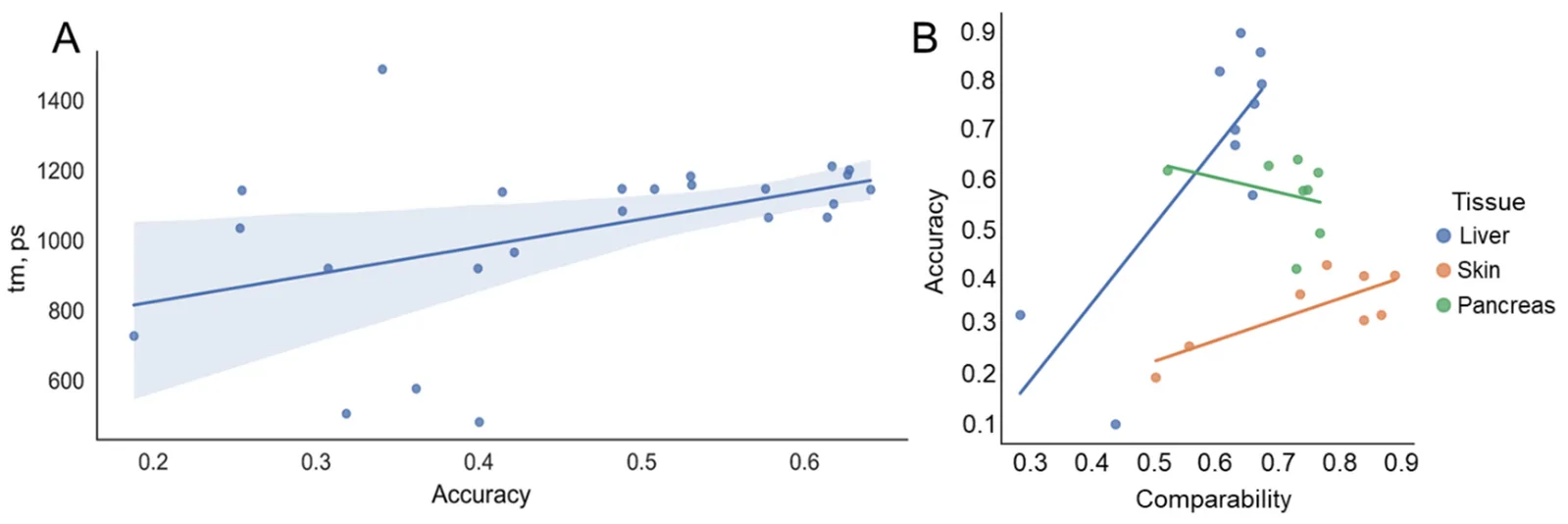
(**A**)—relationship between segmentation accuracy and the value of the weighted average fluorescence decay time, tm. An increase in accuracy corresponds to a decrease in the tm scatter. (**B**)—relationship between segmentation accuracy and comparability.

The lifetime of NAD(P)H determined in the foundational models varies depending on their accuracy. The higher the foundational model accuracy, the more stable the results are compared to manual processing; the scatter is lower. Correlations were shown between accuracy and comparability. A positive correlation was found for liver (0.559) and skin (0.563) tissues, but a negative correlation was found for pancreatic tissue (−0.404). The results are shown in Figure 4.

### 2.4. Classification Accuracy

We developed a classification algorithm for determining normal and pathological conditions for liver, pancreas (islets of Langerhans) and skin tissues.

Parameter a2 was identified as providing the most significant contribution for cell-by-cell analysis, while tm showed the least. For the classification variant based on the values averaged from the individual images, the high significance of the parameters t2 and t3 was shown, while low significance was shown by the parameters a2 and the area of inclusions.

The best-known distinguishing parameter of FLIM is tm. However, since the tm parameter is a weighted average of lifetime parameters and their contributions, it is inherently correlated with all parameters describing fluorescence lifetimes. Moreover, it is known that weighted averages contain less information about the data distribution than about initial parameters, so the latter can also disproportionally affect the results. Therefore, this could influence the outcome of parameter selection during classifier training, thereby limiting its usefulness in the current context. Furthermore, in our earlier studies on FLIM data analysis in liver tissue under pathological and regenerative conditions, we found that the most significant changes occur in the a2 and a3 values [10,11,12,13,18]. Importantly, a2 and a3 are convenient for interpreting the results, as they are directly related to the intensity of various metabolic pathways, including glycolysis, OXPHOS, lipogenesis, and the glutathione cycle.

The algorithm demonstrated high accuracy in classifying individual cells into normal and pathological categories, allowing for a detailed assessment of cellular heterogeneity in the sample. This approach makes it possible to classify samples based on the predominant cellular phenotype.

**Figure 5:**
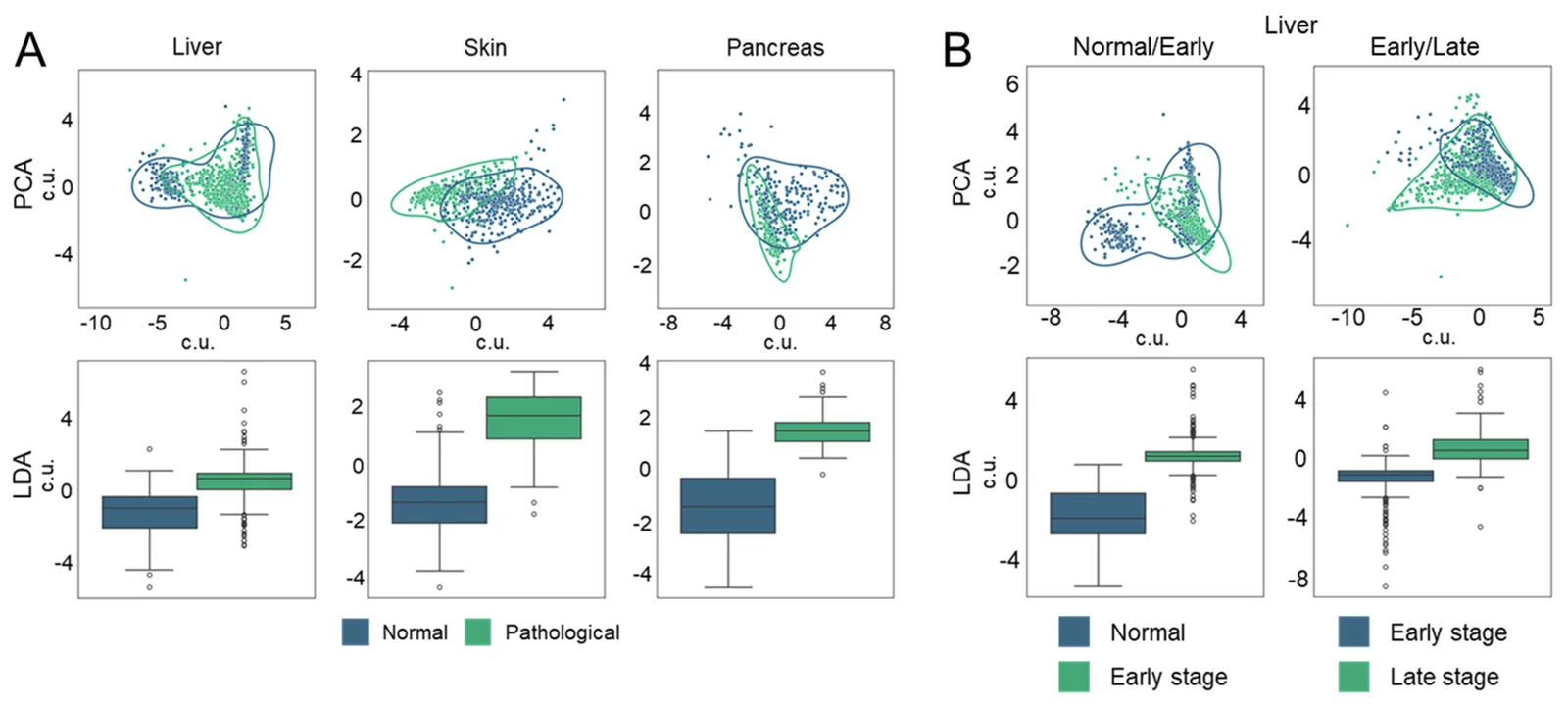
Visualization of mutual distribution for: (**A**)—normal and pathological samples of liver, skin and pancreas tissue; (**B**)—comparison of early and late stages of pathology development of liver tissue.

It has been shown to be highly effective in distinguishing between the early and late stages of liver pathology, as well as in detecting pathology at an early stage. This can make it possible to reveal the presence of pathology even in the early stages of its development, as well as to distinguish the degree of tissue damage during the development of pathology. The corresponding classification accuracy metrics are shown in Table 3, and the mutual distribution of data is illustrated in Figure 5.

High classification accuracy was shown between normal and pathological variants of skin tissue (F1 = 0.95, ROC-AUC (Receiver Operating Characteristic Area Under Curve) = 0.958) and liver (F1 = 0.963, ROC-AUC = 0.964). Relatively high accuracy was shown for the classification of normal and pathological conditions of pancreatic tissue (F1 = 0.836, ROC-AUC = 0.829). At the same time, high accuracy was shown for the classification of normal and early pathological liver tissue (F1 = 0.962, ROC-AUC = 0.963). High accuracy in the classification of variants of early and late pathology (F1 = 0.984, ROC-AUC = 0.984) was also shown. Classification of normal and pathological variants of liver tissue by image was shown with relatively high accuracy (F1 = 0.867, ROC-AUC = 0.844).

## 3. Discussion

Accurate segmentation and classification of tissue images from various anatomical origins represents one of the most significant and challenging applications in biomedical imaging. The development of digital pathology and high-resolution imaging techniques has made automated tissue segmentation essential for accelerating diagnosis, enhancing accuracy, and facilitating large-scale quantitative analyses of diverse tissue types and pathological conditions [38].

In this paper, we developed an automatic classification algorithm to distinguish normal from pathological tissue, thereby creating a rapid decision-support system that simplifies the diagnosis of organ pathologies and reduces human bias. To this end, we employed foundational neural network models for semantic segmentation of fluorescence time-resolved images across multiple tissue types—namely, liver, skin, and pancreas (islets of Langerhans)—in both normal and pathological states. The investigated models were SAM 2, CellposeSAM, LACSS, and CellSAM.

Each segmentation model was tested using a specially developed approach for automatic FLIM data processing. Although most researchers currently rely on manual data processing within commercial software, such an approach suffers from several disadvantages, as outlined in the introduction, including limitations in obtaining the FLIM parameters quickly and without human bias. To address these issues, we developed an automated algorithm to overcome these limitations as follows:

1. Threshold ambiguity has been resolved through automatic image intensity processing, enabling the identification of boundaries between low-, medium-, and high-intensity objects without operator intervention.

2. Selection bias in any region of interest (ROI) definition was addressed by using the foundational models, which perform automatic semantic segmentation by highlighting accessible objects (cells) in the image. For this, when selecting objects across different images, the same mathematical operations are applied, relying solely on object geometry rather than on internal lifetime characteristics.

3. Reproducibility of results is improved because the mathematical functions underlying the automated algorithm constrain the analysis to a predefined methodology. Furthermore, applying any given foundational model to the same images yields identical results regardless of the number of repetitions.

4. Scalability and complex data processing limitations are mitigated by the high processing speed of the automated algorithm.

All studied models can be integrated into various workflows such as spatial transcriptomics and live-cell imaging. They support analysis of 2D images, 3D image stacks, and time-lapse videos, making them convenient for developing custom solutions based on the proposed algorithms—particularly using the widely adopted Python programming language.

We demonstrated that the best combination of quality, speed and comparability of FLIM segmentation is achieved using SAM 2 and LACSS.

However, it should be noted that SAM 2 is the slowest model by a factor of 10 (55.59 s vs. 5.14 s for CellposeSAM). However, the CellposeSAM model yielded mixed results, providing high accuracy only for normal parenchymal tissue. The worst combination of quality, speed, and comparability was observed for the CellSAM model.

Nevertheless, it must be taken into account that, relative to manual processing, FLIM parameter values vary depending on the foundational model used—an effect reflected in the comparability parameter. Importantly, segmentation accuracy was shown to correlate with this comparability parameter: high accuracy corresponds to high comparability values. Therefore, manual quality control of segmentation may still be necessary to preserve the relevant values of the studied FLIM parameters.

Analysis of changes in parameters during manual and automatic processing showed that such changes in values correspond to physiological processes.

Previously, we have identified a relationship between the values of FLIM parameters a2 and a3 and the presence and stage of development of steatosis and fibrosis pathologies. We showed that early stages of pathology development are associated with lower rates of a2 and a3, in comparison with normal tissues, while with the development of pathology, the rates of a2 and a3 increase [10,11,12,13,18].

For skin tissue, melanoma cells exhibit the Warburg effect, shifting metabolism towards glycolysis even in the presence of oxygen, which causes a1 to increase and a2 to decrease, something we can observe when analyzing the images. The parameter tm decreases due to the decrease in the parameter a2, as the short-lived form of NAD(P)H begins to make a larger contribution [39,40,41].

In pancreatic tissue affected by type I diabetes mellitus, no significant differences in the tm parameter were observed between normal and pathological conditions. However, an increase in a2 and decreases in t1 and t2 were noted, likely reflecting pH changes and cytosolic acidification, as well as the induction of compensatory mechanisms including antioxidant defense [14,42,43].

The SAM 2 model has proven its comparability for the segmentation of 3D medical images (such as CT and MRI) as 2D slice sequences [24]. SAM 2 also allows the segmentation of cellular structures, tissue regions, and other microscopic objects in individual frames from biological tissues, including microscopic images, using cues (points, boxes, or masks). The model supports bidirectional segmentation along slices, improving the completeness and accuracy of 3D segmentation by combining forward and backward inferences [44]. It has also been demonstrated that the SAM 2 model can even provide effective segmentation of images of large parenchymatous organs such as the liver, kidneys, and spleen. In particular, CT image segmentation of these organs has been achieved with DSC scores of about 0.82–0.89 in frame zero mode. However, smaller or less distinct structures such as those characteristic of the pancreas, gallbladder, and adrenal glands show lower segmentation accuracy due to weaker boundaries or smaller volumes [45]. In our work, SAM 2 showed high accuracy of cell segmentation in all the studied tissue types, but the data acquisition speed was the slowest among all models. The low segmentation speed is due to the lack of specific attention to segmented objects. This is evidenced by the higher segmentation speeds when using CellSAM and CellposeSAM, which use different methods of representing segmented objects for the first-generation SAM model. It may also be important that SAM 2 was trained mainly on natural images, given that such low-contrast tissue images with layers of identical structures can be difficult to distinguish. Such results are consistent with our data, where we showed under-segmentation for small-cell images of pancreatic tissue. In addition, in our work, there were no segmentation prompts, as the algorithm is aimed at a high degree of automation. However, despite the low speed, we demonstrated that SAM 2 is an effective model for segmenting fluorescence lifetime images.

CellposeSAM adapts the pretrained transformer backbone from the SAM foundation model to the Cellpose ecosystem [26]. This hybrid approach combines SAM’s flexible segmentation capabilities with Cellpose’s generalist algorithms designed for cellular structures. The model significantly outperforms inter-human agreement in segmentation tasks and approaches the human consensus segmentation bound, thereby reducing error rates. In our work, CellposeSAM achieved high segmentation accuracy in homogeneous parenchymal tissue, whereas samples with heterogeneous structures and/or non-parenchymal inclusions exhibited substantially lower accuracy. More specifically, healthy liver tissue exemplifies homogeneously structured tissue, while pathological tissue features lipid infiltration [46] and collagen inclusions [47] that introduce heterogeneity and reduce segmentation efficiency. By contrast, skin and pancreatic tissues are inherently heterogeneous due to their non-parenchymal structure.

Given the U-Net-like architecture of Cellpose [25], it can be assumed that image segmentation quality depends directly on the training material. Thus, despite being trained on a large and diverse dataset (comprising approximately 22,826 images and 3.3 million annotations), CellposeSAM may not fully represent all tissue types or staining modalities [26]. Although the model’s robustness is enhanced through data augmentation techniques—such as channel invariance and noise simulation—tissues exhibiting unusual artifacts, low contrast, or specific staining patterns may still fall outside its training distribution.

The LACSS model combines a location prediction network (LPN) with a fully convolutional network (FCN) for segmentation [27]. The LPN predicts the locations of individual cells (locations of interest or LOIs), while the FCN segmentation focuses on segmenting cells based on these predicted locations [48]. By limiting segmentation computation to a small area around each predicted LOI, LACSS significantly reduces the computational load compared to methods that process entire images. This design facilitates scalability to large datasets and enables higher-throughput microscopy imaging processing. In our work, we found that LACSS consistently achieves moderate to high accuracy, regardless of the type of tissue being segmented. However, we observed some over-segmentation of large cells in liver and skin images, a phenomenon not detected for smaller pancreatic cells. At the same time, LACSS tends to segment cells across the entire image. This behavior may be explained by the fact that LACSS is an anchor-free method that makes no assumptions about cell size or shape [27]; instead, it predicts points of interest and segments only the immediate context around each point.

This local approach makes it robust to varying background complexity. However, while effective in homogeneous or slightly heterogeneous tissues—achieving high accuracy on benchmarks like the Cell Image Library and LIVECell—it can struggle with more complex pathological structures, where boundary detection remains challenging. A key advantage is its flexible training, supporting both full supervision with complete masks and weak supervision using only cell locations or image-level labels, thereby reducing annotation costs without compromising segmentation quality.

CellSAM [29] is a universal foundation model specifically designed for cell segmentation that generalizes across a wide variety of cellular imaging data [49]. The key advantage of CellSAM is its training on a large and diverse dataset spanning multiple cellular archetypes, including tissues, cell cultures, yeast, H&E-stained histological sections, and even bacteria. To automate inference, CellSAM uses an object detector called CellFinder (based on the Anchor DETR transformer framework) that detects cells and generates bounding box prompts that the SAM uses for mask segmentation. A number of studies have shown that CellSAM achieves human-level segmentation accuracy across diverse cells and imaging modalities [29,49]. In our work, CellSAM exhibited sufficiently high segmentation accuracy for the pancreatic tissue, albeit with a substantial degree of under-segmentation of objects. For liver and skin tissue, CellSAM segmented fewer objects compared to manual analysis. Although CellSAM automates cell segmentation via the CellFinder detector, its accuracy was comparable to or lower than that of SAM 2, suggesting limitations in CellFinder’s detection quality. A key issue is that Anchor DETR’s fixed anchor points in dense regions may fall into regions containing multiple cells, leading to query confusion, fused detections, or false positives. Furthermore, the model struggles to segment small cells or nuclei—as observed in pancreatic tissue—due to its reliance on coarse feature maps and an architecture that may not capture fine details effectively [50,51].

Nevertheless, the problem of insufficient segmentation of small objects can be mitigated using a few labeled examples via few-shot learning. The resulting image analysis data can be used to train classifiers—such as logistic regression, Naïve Bayes, support vector machines, and decision trees—to distinguish between normal and pathological conditions.

In our work, we chose to use Decision Trees [52], which offer a rule-based approach for maximum flexibility and intuitive interpretation of the model. This method is based on recursively dividing the feature space using yes/no questions about the features, creating a flowchart-like structure that leads to class prediction. Individual trees are easy to understand and visualize, but they are very sensitive to small changes in data and prone to overfitting. This disadvantage is effectively eliminated by ensemble methods such as Random Forest [53]. This algorithm builds a set of decision trees during training and outputs a class, which is the mode of classes predicted by individual trees. By averaging the results of a variety of uncorrelated trees (achieved using methods such as bagging and random feature selection), Random Forest significantly increases the accuracy and stability of the forecasting, making the approach one of the most powerful and widely used algorithms for structured data. Random Forest has shown high efficiency in the classification of pathologies during probing [8], in analyzing the heterogeneity of tumor tissues [7], and in the classification of brain glioma tumor tissues [9] using FLIM data. Here, we also demonstrated its high accuracy in the classification of pathological metabolic conditions of tissues relative to normal tissues. The other approaches mentioned above had a number of critical shortcomings, as explained below.

Logistic regression predicts class probability by applying a sigmoid function to a linear combination of input parameters. However, it assumes linear relationships among features, which limits its effectiveness for complex, non-linear data [54]. The Naive Bayesian algorithm is based on the calculation of the probability of belonging to a class based on parameter values, with a strong assumption of conditional independence between each pair of features for a given class label, but this is not applicable to FLIM data [55]. Support Vector Machines (SVMs) find an optimal separating hyperplane to maximize class margins and therefore perform well in high dimensions, but they require kernel selection and are most effective when classes are clearly separable [56].

In steatosis, fibrosis, melanoma and diabetes, metabolic differentiations drive tissue heterogeneity through shifts in glycolysis, oxidative phosphorylation (OXPHOS), lipid handling, and amino acid metabolism, adapting cells to pathological stress.

Both steatosis and fibrosis disrupt the native metabolic homeostasis of liver hepatocytes. Under physiological conditions, hepatocytes sustain their diverse functional repertoire—encompassing protein synthesis, lipid metabolism, and xenobiotic detoxification—through high rates of OXPHOS and active biosynthetic pathways [10,11,12,13,18]. In the context of steatosis, this metabolic state of the hepatocytes is altered by the onset of lipotoxicity resulting from intracellular lipid accumulation, which leads to a significant suppression of OXPHOS [57,58,59]. Similarly, fibrosis is characterized by a reduction in OXPHOS activity, primarily attributable to portal hypoxia caused by the impaired delivery of oxygen and nutrients to the parenchyma [60,61].

For skin tissue and melanoma cells, normal skin exhibits metabolic homeostasis with keratinocytes relying on glycolysis for proliferation and differentiation, fibroblasts on OXPHOS for extracellular matrix maintenance, and melanocytes using low-rate glycolysis coupled with melanin synthesis via tyrosinase. In melanoma progression, tumor cells undergo a Warburg shift to aerobic glycolysis, upregulating GLUT1, HK2, LDHA, and HIF1α for rapid proliferation and lactate export, creating acidic microenvironments that suppress immune responses. Melanoma subpopulations diverge metabolically: glycolytic clusters drive invasion while oxidative ones (high PGC1α, mitochondrial biogenesis) confer therapy resistance and metastasis; normal melanocytes lack this plasticity [17,62,63].

In the case of pancreatic tissue and islets of Langerhans, the stress and dysfunction of islet cells in the course of pancreatic diseases have a direct impact on their energy metabolism. Specifically, they result in a reshuffling of the glucose metabolic pathways. It has been previously demonstrated that in healthy β-cells, an increase in glucose concentration leads to a corresponding rise in ATP levels within the cells through OXPHOS. Conversely, in pathological β-cells, there is an increased occurrence of glycolysis [14,64,65].

## 4. Materials and Methods

To compare the segmentation algorithms and develop an automatic approach for processing FLIM data, we used tissue samples, including liver, pancreas (islets of Langerhans), and skin, both in pathological and normal states. All tissue samples as well as the FLIM data were collected at the Privolzhsky Research Medical University.

For the liver tissue, 31 images of normal tissue, 37 images of fibrosis and 36 images of steatosis were analyzed; for skin tissue, 4 images of each of normal and pathology were analyzed, and for pancreatic tissue, 3 images of normal and 3 of pathology were analyzed.

### 4.1. Experimental Models

#### 4.1.1. Liver

The experiments were performed on male Wistar rats weighing 250–300 g. The rats were divided into three groups—healthy animals with normal liver and animals with either induced hepatic steatosis or fibrosis. Fibrosis was induced by intraperitoneal administration of 30% CCl4 dissolved in oil (n = 10). Steatosis was induced by a high-fat diet, with 60% of the animals’ diet consisting of fat (n = 10). Changes were monitored by collection of liver tissue at 3, 6, 9, and 12 weeks in the case of steatosis, and at 2, 4, 6, and 8 weeks in the case of fibrosis. Normal liver tissue was also collected from the healthy rats (n = 10). The study is described elsewhere [18].

#### 4.1.2. Islets of Langerhans

An experiment was carried out on male Wistar rats weighing 200–230 g. Normal islets of Langerhans were obtained from healthy rats (n = 3).

Diabetes mellitus type I (DM) was induced by intraperitoneal injection of streptozotocin (STZ) at 65 mg/kg of body weight. DM was confirmed by measurements showing blood glucose concentrations greater than 16.7 mmol/L using a glucometer (AccuChek Active; Roche Diabetes Care, Bern, Switzerland). The experiment lasted 3 months, with subsequent isolation of the islets of Langerhans. The study is described in more detail in [14,66].

#### 4.1.3. Melanoma Tumor

Non-lesional skin tissue samples were obtained from healthy patients (n = 3). Melanocytic lesions of 3 patients in the Privolzhsky Research Medical University Clinic were included in the study. A routine dermoscopy examination was followed by calculation of the total dermoscopy score (TDS). Invasive melanoma lesions (n = 3) had TDS ≤  4.75. The study is described in more detail in [17].

### 4.2. FLIM

The liver and pancreas tissue samples were examined using a confocal laser scanning microscope, the LSM 880 (Carl Zeiss, Oberkochen, Germany), equipped with a femtosecond Ti:Sapphire laser Mai Tai^®^ HP (Spectra-Physics, Milpitas, CA, USA) operating at an 80 MHz repetition rate and with a pulse duration of less than 100 fs. The average power incident on any sample was less than 10 mW. The epifluorescence signal of Nicotinamide Adenine Dinucleotide (NAD(P)H) was excited at 750 nm and detected in the range from 450 nm to 490 nm using a bandpass filter (470/40 BP, Chroma, Bellows Falls, VT, USA) and a shortpass filter (690 SP, Chroma, Bellows Falls, VT, USA). A single-photon counting hybrid detector, the HPM-100-40 (Becker & Hickl GmbH, Berlin, Germany), was coupled to the side port of the microscope and an SPC-150NX (Becker & Hickl GmbH, Berlin, Germany) photon-counting card was used to collect the photon statistics. FLIM images were acquired through an oil immersion objective, the C Plan-Apochromat 40×/1.30 (Carl Zeiss, Oberkochen, Germany). The images were 512 × 512 pixels and 212 × 212 μm in size, using a 1024-time channel. The acquisition time of a whole FLIM image was 60 s at a 1 µs pixel dwell time [18].

All skin lesions were examined using an MPTflex™ (JenLab, Berlin, Germany) multiphoton fluorescence tomograph, equipped with a single-photon detector, the PMC-100, and a TCSPC SPC 150 photon counting card (both from Becker & Hickl GmbH, Berlin, Germany) to perform fluorescence decay measurements. A femtosecond tunable Ti:sapphire laser (repetition rate 80 MHz, pulse duration less than 100 fs), MaiTai^®^ (Spectra-Physics, Milpitas, CA, USA), working at a wavelength of 750 nm, was used to excite epifluorescence of the reduced form of NAD(P)H. The average laser power used was less than 10 mW. Emission was recorded in the spectral range of 409–660 nm. An oil immersion objective, the EC Plan-Neofluar 40x/1.30 (Carl Zeiss, Oberkochen, Germany), was used to acquire images (127 × 127 pixels, 230 × 230 μm in size, using a 256-time channel). Image acquisition time was 6 s [17].

### 4.3. Manual FLIM Data Processing

The manual FLIM analysis was performed using SPCImage 8.3 software (Becker & Hickl GmbH, Berlin, Germany) with a bi- or tri-exponential decay model. To provide reasonable S/N ratios, typically ~5000 and 10,000 photons were collected for the bi-exponential and tri-exponential models, respectively. The goodness-of-fit of the model was assessed using the χ^2^ values, which were in the range of 0.9–1.2. The FLIM parameters were analyzed in 20–30 regions of the cell cytoplasm for each field of view. In the case of the bi-exponential model, the FLIM parameters analyzed were the amplitude-weighted mean fluorescence lifetimes (tm) and the relative contributions of the free, a1 (%), and the bound, a2 (%), forms of NAD(P)H. In the case of the tri-exponential model, the relative contribution of NADPH, a3 (%), was added to the analysis.

The formula for the mean fluorescence decay time is:

$$t_{mean}=\frac{\sum a_{n}*t_{n}}{\sum a_{n}}.$$

Tri-exponential decay approximations were used for the liver tissue analysis, while bi-exponential ones were used for both the skin and pancreas tissue analyses. This choice was made based on the literature and practical results given in articles regarding the manual analysis of selected tissues 13, 15, 17.

For liver tissue, we collected 10 images from normal specimens and 20 images from both steatotic and fibrotic samples. For the islets of Langerhans, we collected 20 images from normal specimens and 20 images from diabetic islets. For skin, we collected 10 images from healthy patients and 10 images from patients with melanoma lesions.

For each image included in the study, cell mask segmentation was performed manually, and the maximum available number of cells was allocated, on average, 15–25. The analysis was performed in ImageJ 1.54p (National Institutes of Health, Bethesda, MD, USA).

### 4.4. Automatic FLIM Data Processing

The most common foundational models for tissue or organ segmentation in biomedical imaging were selected for the analysis: CellSAM, CellposeSAM, LACSS and SAM. All of these models have open repositories on GitHub (https://github.com/). The selected segmentation models can be integrated into the image processing algorithm and do not require mapping or labeling to perform semantic segmentation.

We developed an algorithm for automatic preprocessing and segmentation of the studied fluorescence images with extraction of the FLIM parameters, which made it possible to quickly obtain and record cell-by-cell data.

Firstly, the images were processed in SPCImage, using an approximation based on the function of interest. For liver tissue, decay curves were approximated based on the tri-exponential model, with a fixed value of the fluorescence lifetime of the free form of NADH (t1) equal to 400 ps. For skin and pancreatic tissues, we used the bi-exponential approximation model. After image processing, the obtained maps of the parameters of interest were exported, including a1, a2, a3, tm, and chi.

The raw images were converted into intensity images, where each pixel value is the number of recorded photons. The intensity images were then converted to a uint8 format suitable for image modification using Python libraries. To equalize the intensity of the images, we used an adaptive histogram equalization algorithm from the cv2 library, followed by kernel convolution to increase sharpness. Finally, Min-Max normalization was applied to the resulting images for further application of convolutional neural networks, if necessary.

A two-threshold Otsu algorithm was applied to the images to extract the intensity boundaries for excessively bright objects corresponding to various inclusions (e.g., vitamin A, melanin) and excessively dark ones corresponding to the background of the image, to breaks and to cell nuclei. Based on the intensity boundaries, we obtained masks of objects that were subsequently excluded from the studied images. Thus, the images became suitable for processing by the foundational models.

The foundational model libraries used have been downloaded and installed in a workspace based on Python (3.13.9). To obtain segmented masks using the CellSAM and CellposeSAM models, we used the standard hyperparameters of these foundational models. For LACSS and SAM 2, we selected hyperparameters that provided efficient segmentation based on the quality of the segmentation mask and on accuracy parameters. The parameter selection was carried out on a limited number of images of varying quality. For SAM 2, mask_threshold, pred_IoU_threshold, and stability_score_threshold were selected, while for LACSS, the detection_threshold, segmentation_threshold and nms_threshold were used. A visual assessment of various mask variants obtained based on different hyperparameters was carried out, and the most effective parameters were selected—those that allowed identification of the largest number of objects.

Based on the resulting final masks, the FLIM parameters for each segmented object and for the relevant cells were extracted from the maps of the parameters previously gathered using SPCImage. The received FLIM parameters for each individual object were recorded.

### 4.5. Comparison of Foundational Models

In total, the number of objects of interest segmented by each of the different models was as follows: CellSAM—1309, SAM 2—2074, CellposeSAM—4296, LACSS—6240. Manual processing of 912 objects of interest took place.

For each segmentation model, we determined the quality, speed and comparability of FLIM image segmentation.

To evaluate the accuracy of automatic segmentation, we compared manually obtained cell-by-cell masks with the automatic segmentation models.

Quality: the quality of segmentation was analyzed by comparing masks obtained for images of normal and pathological tissues, in order to assess the effect of different degrees of tissue structure on the results of segmentation. The segmentation accuracy was estimated on the basis of no more than 100 labeled cells for each tissue variant. The cells in the image were selected randomly, without a detailed manual description, due to the lack of qualified personnel.

To compare the quality of segmentation, a range of statistical indicators was used: Precision, Recall, F1-score (Dice score), Jaccard score (IoU) and Average Precision (AP).

Precision quantifies the accuracy of positive predictions. It is defined as the ratio of true positive instances to the total number of instances predicted as positive.

Precision = TP/(TP + FP)

Recall measures the ability of a model to identify all relevant positive instances. It is defined as the ratio of true positive instances to the total number of actual positive instances.

Recall = TP/(TP + FN)

The F1-score is the harmonic mean of precision and recall, providing a single metric that balances the trade-off between them. It is mathematically equivalent to the Sørensen–Dice coefficient, a similarity measure commonly used in image segmentation.

F1 (Dice) = 2 ∗ (Precision ∗ Recall)/(Precision + Recall)

The Jaccard score, or Intersection over Union (IoU), measures the similarity between finite sample sets. It is defined as the size of the intersection divided by the size of the union of the predicted and ground truth sets.

Jaccard (IoU) = |A ∩ B|/|A ∪ B|

Average Precision (AP) is a performance metric that summarizes the shape of the precision-recall curve into a single score.

All the presented parameters are important indicators of segmentation quality. But, in our case, we could not compare masks by area for each individual cell, since the masks obtained manually were based on a random selection of cells. The most suitable option for comparing masks derived by manual marking and masks obtained automatically is the use of Recall, which allows estimation of the area of intersection between the automatically obtained mask and its manually created counterpart. The quality of the automatically obtained masks was assessed manually by experienced histologists.

The remaining segmentation accuracy indicators, in turn, make it possible to control the quality of the automatic masks, but do not allow us to compare them directly.

Speed: the segmentation speed was measured directly using the Python time (version 3.7) plugin, which measures the duration of the segmentation process.

Comparability: The data obtained by manual processing in SPCImage were used as a control for comparing all automatic segmentation models. Parameters a2, a3 and tm were chosen for evaluating the comparability, since a2 is largely inversely proportional to parameter a1 (the correlation is −0.882), while a3 has a weak correlation of 0.484 with a2. The tm parameter is a weighted average of the parameters t1, t2, t3, with weights of a1, a2, a3.

To compare the segmentation provided by the different foundational models for liver tissue in normal and pathological states, we assessed the mutual correlation of the distribution of FLIM parameters (a2, a3 and tm) for both the manual and automatic processing options. Collected data for different conditions were compared with each other using the Dunn test, adjusted for multiple comparisons with Benjamini–Hochberg adjustment, to obtain matrices of the relationship between the various pathology stages. The resulting matrices were compared with those for the manual processing option to obtain an error matrix for each comparison metric (Accuracy, Precision, Recall, F1 and ROC-AUC), which allowed us to assess the degree of similarity between the distributions of the FLIM parameter values obtained using the different models of automatic segmentation.

To analyze the effectiveness of image segmentation of skin and pancreatic tissue in both normal state and with pathology, we chose a sample comparison method based on the Kolmogorov–Smirnov (KS) test. FLIM parameter values (a2 and tm) for the normal and pathological tissue obtained by manual processing were compared with the corresponding values obtained by automatic segmentation to determine the KS statistics. 1—the samples are identical; 0—the samples are different. To derive a single parameter for each type of specimen, the harmonic mean was calculated.

The metrics of accuracy and comparability complement each other conceptually, enabling efficient distinction between the different variants of automated analysis. Since the segmentation model may provide high geometric accuracy (perfectly delineating cell boundaries), but at the same time generate parameter distributions that systematically differ from the results of manual analysis (low comparability), or, conversely, may provide moderate geometric accuracy but still preserve the statistical properties of the FLIM parameters for different cell populations, both indicators are necessary: Accuracy ensures the technical correctness of the segmentation, while comparability confirms that the biological interpretation aligns with generally accepted manual analysis methods.

Visual Studio Code (1.103, Microsoft, Redmond, WA, USA) was used as the analysis environment, and Python (3.13.9, Python Software Foundation, Wilmington, DE, USA) was used as the programming language.

### 4.6. Classification Analysis

The most effective variants of pre-trained segmentators were used as the segmentation method for the corresponding tissue variants: LACSS for both liver and skin tissue, and CellSAM for pancreatic tissue.

For liver tissue, we isolated and obtained 9834 individual decay values (of which 2952 were normal values, 3307 were values for various stages of fibrosis development, and 3575 were values for various stages of steatosis development). For skin tissue, 738 separate fluorescence decays were obtained (of which 390 were normal values and 348 were for melanoma). In the case of pancreatic tissue, 420 separate values were obtained (of which 210 were normal values and 210 were for melanoma).

Therefore, to automatically differentiate normal and pathological tissue, the classification models were developed using an ensemble Random Forest algorithm with automatic hyperparameter selection. This algorithm was chosen because, with a small branching depth (up to 3), it is possible to obtain a sufficiently high-precision result and, at the same time, generate a decision tree that can be used for manual analysis using a trained algorithm, without the use of machine learning methods.

Data obtained from parameter maps using the LACSS method for liver and skin tissue, and CellSAM for pancreatic tissue, were used for training. Separate predictive models were trained for each of the three tissue types. The training set consisted of 50% of the cellular dataset. The relative importance of kinetic parameters (a1, a2, a3, t2, t3, and tm) in distinguishing between normal and pathological conditions was assessed.

The classification efficiency analysis was carried out on the basis of a validation sample. For each classifier variant, the results for one or more images corresponding to the results of the norm or pathology were taken from the set of training and test data. The results of these images were further used to analyze the classification efficiency.

A classification algorithm was trained to detect different groups based on cell-by-cell data previously obtained using the developed image analysis algorithm. An analysis of the classification results was also carried out based on the average data variants obtained for each individual image. The results included the median values of the fluorescence decay parameters, their variations [Q3–Q1], as well as the values of the areas occupied by bright inclusions.

The differences between the normal and pathological conditions for liver, skin, and pancreatic tissues were analyzed. The differences between normal liver tissue and the early development of pathology, and the early and late stages of development of liver tissue pathology, were also analyzed.

Sensitivity, Specificity, Precision, Recall, F1, and ROC-AUC were used as classification quality metrics. The classifiers were trained, and accuracy was evaluated without random seed control. Multiple repetitions of the resulting values were shown for all variants, for different training variants and for the test samples.

Dimensionality reduction methods were used to provide visualization of the mutual distribution of classified groups: principal component analysis (PCA) and linear discriminant analysis (LDA).

## 5. Conclusions

We have developed an effective decision-making system for the rapid differentiation of normal and pathological tissues based solely on FLIM data using a Random Forest classifier. This classifier leverages the pronounced metabolic profile changes characteristic of pathological conditions to effectively differentiate between them. We also identified the most effective model for image segmentation by testing a range of widely used segmentation models on both simple-structured normal tissues and complex pathological tissue structures. The SAM 2 model represents a good option from the perspective of segmentation quality, owing to the high generalization ability inherent to its algorithm. As a result, even zero-shot analysis achieves fairly high quality without the need for prompts. However, SAM 2 is the slowest option, and difficulties may arise when segmenting small-cell variants, such as in pancreatic tissue. The LACSS and CellposeSAM models and their modifications have high accuracy and allow accurate detection of cells, but are very dependent on the training material, which, for obvious reasons, did not include FLIM images. The CellSAM model produced low-quality segmentation, except in small-cell areas of the pancreatic tissue, and consistently under-segmented all tissue types. This decline in performance likely stems from the Cellfinder module (the key difference from SAM), which was trained on a limited cellular and tissue image dataset, thereby restricting its performance relative to other models.

Automated algorithms for the classification of pathological tissues based on metabolic differentiation using fluorescence lifetime analysis can improve high-precision diagnostics. Application of the algorithm we have developed can be scaled to various tissue types and pathologies, as supported by evidence-based analysis of its effectiveness. The combination of automated image analysis with classification methods makes it possible to significantly increase the sample size under study and therefore to obtain accurate results by significantly expanding both the training and experimental datasets.

## Figures and Tables

**Table 1:** Comparative analysis of four models for image segmentation—SAM 2, CellposeSAM, LACSS and CellSAM.

Feature	SAM 2 [24]	CellposeSAM [26]	LACSS [27]	CellSAM [29]
**Core Architecture**	Vision Transformer (ViT) + fine-tuned decoder	Cellpose spatial gradients + SAM prompting	LPN + FCN	SAM + CellFinder detector (Anchor DETR [30])
**Training Approach & Data**	Fine-tuned on microscopy datasets (e.g., LIVECell [31])	Combines Cellpose pretraining with SAM; uses cell culture/tissue data	Cell Image Library [32] and LIVECell	Trained on 1M+ annotations (TissueNet [33], Cellpose [34], DSB [35], DeepBacs [36])
**Automation Level**	Semi-automatic—may require prompts; AMG struggles with dense cells	Semi-automatic—human-in-the-loop tuning recommended, but not necessary	Fully automatic—grid-point prompts, no human input	Fully automatic—built-in CellFinder detector generates prompts
**Performance Metrics**	Dice: 0.92 (LIVECell); improves with fine-tuning	Dice: 0.87 (combined dataset)	mAP (mean average precision) 0.5:0.81 across 19 datasets; excels in cross-domain tasks	mAP 0.5:0.84 (MoNuseg [37])
**Computational Demand**	High (ViT-L configuration); GPU-intensive	Moderate; slower than Cellpose alone (dual-model pipeline)	Moderate	Moderate
**Tissue-Specific Suitability**	Requires fine-tuning; struggles with packed cells	Generalist; adapts to tissues via Cellpose backbone	Robust against morphology shifts; ideal for novel tissues	Optimized for histology/tissues (TissueNet-trained)
**FLIM Performance**	Best overall accuracy; slowest (55.6 s); excels at large structures	High accuracy for homogeneous tissue; variable for heterogeneous	Consistent moderate-high accuracy; over-segmentation of large cells	Best for small cells (pancreas); under-segmentation for others
**GitHub**	https://github.com/facebookresearch/sam2 (accessed on 2 March 2026)	https://github.com/MouseLand/cellpose (accessed on 2 March 2026)	https://github.com/jiyuuchc/lacss (accessed on 2 March 2026)	https://github.com/vanvalenlab/cellSAM (accessed on 2 March 2026)

**Table 2:** Comparative analysis of segmentation models’ accuracy and comparability.

Model	Liver		Skin		Pancreas	
	Normal	Pathological	Normal	Melanoma	Normal	Diabetis
Accuracy						
SAM 2	0.51 ± 0.306	0.543 ± 0.255	0.341 ± 0.146	0.398 ± 0.087	0.856 ± 0.032	0.665 ± 0.163
CellposeSAM	0.644 ± 0.032	0.463 ± 0.038	0.429 ± 0.222	0.17 ± 0.126	0.446 ± 0.381	0.463 ± 0.105
LACSS	0.703 ± 0.045	0.679 ± 0.034	0.711 ± 0.068	0.822 ± 0.075	0.609 ± 0.053	0.566 ± 0.059
CellSAM	0.25 ± 0.097	0.181 ± 0.126	0.271 ± 0.352	0.413 ± 0.198	0.937 ± 0.015	0.743 ± 0.087
Comparability						
SAM 2	0.663	0.66	0.84	0.868	0.686	0.523
CellposeSAM	0.641	0.675	0.78	0.504	0.731	0.769
LACSS	0.632	0.633	0.84	0.89	0.742	0.749
CellSAM	0.285	0.436	0.558	0.737	0.733	0.766

**Table 3:** Classification accuracy metrics.

	Tissue					
Metric	Liver (Normal/Late)	Liver (Normal/Early)	Liver (Early/Late)	Liver (by Image)	Skin	Pancreas
Sensitivity	0.843	0.85	0.991	0.867	1	0.859
Specificity	1	1	0.977	0.837	0.905	0.803
Precision	1	1	0.977	0.837	0.905	0.803
Recall	0.929	0.927	0.99	0.9	1	0.871
F1	0.963	0.962	0.984	0.867	0.95	0.836
ROC-AUC	0.964	0.963	0.984	0.844	0.958	0.829

## Data Availability

The results of manual and automated analysis using the developed algorithm are available in the repository at the link: https://doi.org/10.5281/zenodo.19678566. The raw FLIM data presented in this study are available on request from the corresponding authors.

## Supplementary Materials

The following supporting information can be downloaded at: https://www.mdpi.com/article/10.3390/ijms27188162/s1.
