# Supplementary material for: Neural Network-Based System for Rapid Diagnostic Decision Support Using FLIM Data: Segmentation and Classification in Liver, Skin, and Pancreatic Tissues
Source: Int J Mol Sci. 2026 Sep 14;27(18):8162. doi: 10.3390/ijms27188162 (PMC13607095; doi:10.3390/ijms27188162)
Supplement: Supplementary file 1 [file ijms-27-08162-s001.zip › ijms-4509958-supplementary.pdf]

Supplementary materials

Table S1. Comparison of segmentation models for normal and pathologic liver tissue images.

Methods	Precision	Recall	F1(Dice)	Jaccard(IoU)	AP	HM
Normal						
SAM 2	0.731 ± 0.116	0.51 ± 0.306	0.562 ± 0.273	0.435 ± 0.277	0.632 ± 0.136	0.53 ± 0.205
CellposeSAM	0.755 ± 0.142	0.644 ± 0.032	0.655 ± 0.049	0.551 ± 0.027	0.691 ± 0.037	0.626 ± 0.036
LACSS	0.709 ± 0.018	0.703 ± 0.045	0.6895 ± 0.009	0.54 ± 0.011	0.64 ± 0.011	0.617 ± 0.01
CellSAM	0.647 ± 0.002	0.25 ± 0.097	0.345 ± 0.099	0.241 ± 0.089	0.569 ± 0.056	0.341 ± 0.077
Pathologic						
SAM 2	0.664 ± 0.106	0.543 ± 0.255	0.556 ± 0.172	0.424 ± 0.166	0.574 ± 0.076	0.508 ± 0.118
CellposeSAM	0.708 ± 0.222	0.463 ± 0.038	0.514 ± 0.066	0.406 ± 0.041	0.588 ± 0.06	0.488 ± 0.053
LACSS	0.597 ± 0.015	0.679 ± 0.034	0.61 ± 0.017	0.459 ± 0.01	0.546 ± 0.009	0.531 ± 0.01
CellSAM	0.505 ± 0.245	0.181 ± 0.126	0.255 ± 0.164	0.174 ± 0.12	0.493 ± 0.06	0.254 ± 0.095

Table S2. Comparison of segmentation models for normal and melanoma skin tissue images.

Methods	Precision	Recall	F1(Dice)	Jaccard(IoU)	AP	HM
Normal						
SAM 2	0.506 ± 0.101	0.341 ± 0.146	0.35 ± 0.048	0.232 ± 0.043	0.385 ± 0.02	0.307 ± 0.031
CellposeSAM	0.508 ± 0.195	0.429 ± 0.222	0.435 ± 0.196	0.355 ± 0.157	0.5 ± 0.98	0.422 ± 0.138

LACSS	0.41 ± 0.053	0.711 ± 0.068	0.5 ± 0.027	0.348 ± 0.03	0.38 ± 0.035	0.399 ± 0.03
CellSAM	0.383 ± 0.144	0.271 ± 0.352	0.261 ± 0.272	0.187 ± 0.21	0.374 ± 0.093	0.253 ± 0.156
Melanoma						
SAM 2	0.421 ± 0.021	0.398 ± 0.087	0.369 ± 0.042	0.253 ± 0.034	0.362 ± 0.029	0.318 ± 0.034
CellposeSAM	0.319 ± 0.263	0.17 ± 0.126	0.187 ± 0.128	0.132 ± 0.093	0.328 ± 0.072	0.188 ± 0.092
LACSS	0.382 ± 0.089	0.822 ± 0.075	0.509 ± 0.095	0.357 ± 0.086	0.367 ± 0.085	0.4 ± 0.089
CellSAM	0.458 ± 0.23	0.413 ± 0.198	0.414 ± 0.207	0.298 ± 0.173	0.395 ± 0.144	0.361 ± 0.171

Table S3. Comparison of segmentation models for normal and diabetes pancreas (islets of Langerhans) tissue images.

Methods	Precision	Recall	F1(Dice)	Jaccard(IoU)	AP	HM
Normal pancreas						
SAM 2	0.629 ± 0.042	0.856 ± 0.032	0.708 ± 0.035	0.581 ± 0.041	0.607 ± 0.036	0.628 ± 0.037
CellposeSAM	0.537 ± 0.174	0.446 ± 0.381	0.437 ± 0.281	0.331 ± 0.255	0.519 ± 0.111	0.414 ± 0.182
LACSS	0.724 ± 0.088	0.609 ± 0.053	0.636 ± 0.052	0.5 ± 0.03	0.611 ± 0.054	0.576 ± 0.042
CellSAM	0.62 ± 0.083	0.937 ± 0.015	0.731 ± 0.058	0.599 ± 0.075	0.607 ± 0.076	0.641 ± 0.069
Pancreas with diabetes mellitus						
SAM 2	0.748 ± 0.08	0.665 ± 0.163	0.67 ± 0.074	0.527 ± 0.082	0.683 ± 0.031	0.618 ± 0.052
CellposeSAM	0.711 ± 0.058	0.463 ± 0.105	0.52 ± 0.066	0.377 ± 0.064	0.625 ± 0.023	0.488 ± 0.041
LACSS	0.765 ± 0.036	0.566 ± 0.059	0.629 ± 0.038	0.474 ± 0.04	0.669 ± 0.026	0.578 ± 0.033

CellSAM	0.665 ± 0.083	0.743 ± 0.087	0.685 ± 0.064	0.538 ± 0.083	0.638 ± 0.061	0.614 ± 0.068

Table S4. Comparison of comparability with manual analysis for normal liver tissue images.

	a2 (1-KS)	a3 (1-KS)	tm (1-KS)	HM
Normal liver				
SAM2	0.814	0.51	0.748	0.663
cellposeSAM	0.762	0.462	0.831	0.641
LACSS	0.69	0.485	0.807	0.632
cellSAM	0.207	0.342	0.359	0.285

Table S5. Comparison of comparability with manual analysis for pathological liver tissue images.

Methods	Accuracy	Precision	Recall	F1(Dice)	ROC-AUC	HM
Steatosis						
SAM2	0.66 ± 0.09	0.507 ± 0.171	0.57 ± 0.279	0.531 ± 0.223	0.636 ± 0.13	0.579 ± 0.164
CellposeSAM	0.612 ± 0.036	0.472 ± 0.078	0.784 ± 0.267	0.586 ± 0.139	0.64 ± 0.078	0.611 ± 0.1
LACSS	0.578 ± 0.056	0.446 ± 0.093	0.786 ± 0.263	0.567 ± 0.145	0.58 ± 0.089	0.573 ± 0.111
CellSAM	0.667 ± 0.049	0.645 ± 0.093	0.246 ± 0.023	0.354 ± 0.019	0.581 ± 0.019	0.44 ± 0.02
Fibrosis						
SAM2	0.733 ± 0.067	0.629 ± 0.125	0.886 ± 0.103	0.731 ± 0.101	0.751 ± 0.052	0.741 ± 0.069
CellposeSAM	0.711 ± 0.101	0.61 ± 0.066	0.952 ± 0.083	0.74 ± 0.036	0.74 ± 0.113	0.74 ± 0.054
LACSS	0.644 ± 0.102	0.556 ± 0.127	0.952 ± 0.083	0.692 ± 0.09	0.693 ± 0.046	0.692 ± 0.061

CellSAM	0.578 ± 0.039	0.478 ± 0.135	0.305 ± 0.116	0.371 ± 0.125	0.536 ± 0.047	0.438 ± 0.068

Table S6. Comparison of comparability with manual analysis for normal and pathological liver skin and pancreas images.

	a2 (1-KS)	tm (1-KS)	HM
Normal skin			
SAM2	0.855	0.826	0.84
cellposeSAM	0.772	0.788	0.78
LACSS	0.835	0.845	0.84
cellSAM	0.608	0.515	0.558
Melanoma skin			
SAM2	0.87	0.866	0.868
cellposeSAM	0.48	0.531	0.504
LACSS	0.92	0.862	0.89
cellSAM	0.73	0.745	0.737
Normal pancreas			
SAM2	0.8	0.601	0.686
cellposeSAM	0.758	0.705	0.731
LACSS	0.786	0.703	0.742
cellSAM	0.75	0.717	0.733
Pancreas with diabetes mellitus			
SAM2	0.449	0.625	0.523
cellposeSAM	0.702	0.849	0.769
LACSS	0.678	0.837	0.749
cellSAM	0.695	0.852	0.766
